# Development and applications of artificial symmetrical proteins

**DOI:** 10.1016/j.csbj.2020.10.040

**Published:** 2020-11-27

**Authors:** Jeroen P.M. Vrancken, Jeremy R.H. Tame, Arnout R.D. Voet

**Affiliations:** a Laboratory of Biomolecular Modelling and Design, Department of Chemistry, KU Leuven, Celestijnenlaan 200G, 3001 Leuven, Belgium; b Graduate School of Medical Life Science, Yokohama City University, 1-7-29 Suehiro, Yokohama, Kanagawa 230-0045, Japan

**Keywords:** Symmetry, Protein design, Artificial proteins, Tandem repeats, Solenoid proteins, Toroid proteins, Biotechnology, Structural biology

## Abstract

Since the determination of the first molecular models of proteins there has been interest in creating proteins artificially, but such methods have only become widely successful in the last decade. Gradual improvements over a long period of time have now yielded numerous examples of non-natural proteins, many of which are built from repeated elements. In this review we discuss the design of such symmetrical proteins and their various applications in chemistry and medicine.

## 1 Introduction

**Fig. 1:**
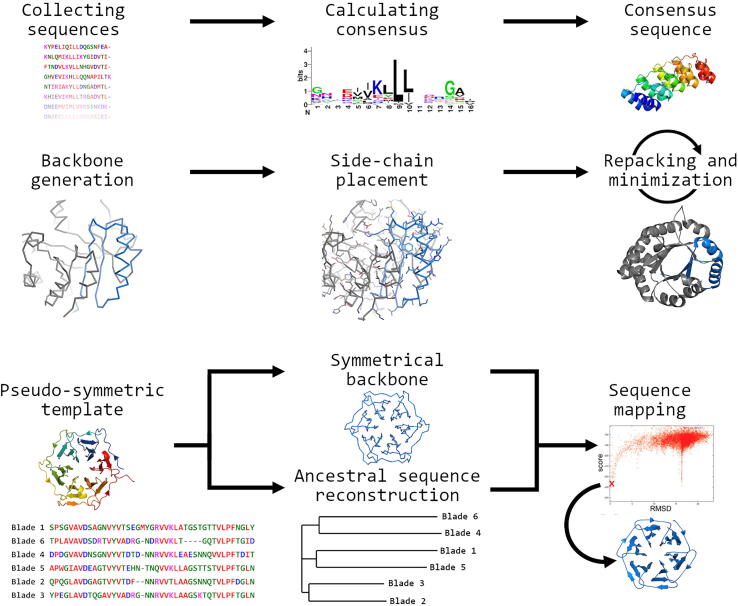
Schematic representation of symmetric protein design. Consensus-based design (top row) starts with the alignment of sequences. Highly variable, non-conserved residues can be chosen manually. Backbone design (second row) is required for the de novo design of perfectly symmetrical structures, and structural constraints may be used to fit a backbone into a specific desired fold. Side-chains are mapped onto the optimized backbone and then the side-chains are packed and energy-minimized in repeated cycles. The bottom row shows the RE_3_volutionary design strategy used to design the Pizza protein. Starting from a pseudo-symmetric template protein sequence, the sub-domain sequences are aligned and putative ancestral sequences are generated. A perfectly symmetric backbone is made from the template model, and the possible ancestral sequences are mapped onto it. Each resulting model is then scored using an energy function to select the most promising sequences.

Symmetry has played an important role in the development of many ideas and disciplines, ranging from the building of ancient Greek temples to Kepler’s mathematical description of the solar system. More recently it has proved central to theories describing subatomic particles and analysis of spatial interactions between objects of all scales. Symmetry turns out to be very useful in constructing almost anything, and even the earliest civilizations preferred symmetrical bricks to irregular building blocks. Nowadays, this preference is reflected in molecular assemblies, which are often built around symmetry axes in two- and three-dimensions [1]. Symmetrical construction is such a universal principle that the first model to be determined of myoglobin surprised Kendrew and Perutz for its irregularity, but some decades later it is now clear that a majority of proteins assemble into symmetrically arranged oligomers, with dimers being the most common quaternary form [2]. Analysis of known protein structures with CE-symm, a bio-informatic program designed to search for internal symmetry in models, shows that about a quarter of all monomeric proteins are assembled from repeated sequence motifs [3]. Almost a fifth of the superfamilies identified by SCOP were found to be pseudo-symmetric, including superfolds such as trefoils, TIM-barrels, and β-propeller proteins [4], [5]. Members of these families may have surprisingly well obeyed structural pseudo-symmetry, even if to the eye the sequence of the consensus motif is not notably conserved. This tendency of evolution to favor internal and external symmetry arises from the energy minimization achieved by symmetrical assemblies [6]. From the very nature of DNA replication, there is always a small chance of sequences becoming duplicated into tandem copies, and copying errors are more likely to link two neighboring DNA sequences within a genome than distant parts. Over long expanses of time therefore genes will appear with internal repeats, and these have apparently formed the basis for many natural proteins in the world around us. While gene duplication and fusion events can lead to symmetrical frameworks, the resulting gene is also subject to genetic drift, allowing it to develop a function, such as a binding property, that requires local symmetry to be broken. The sequence of such proteins, and especially the surface, may therefore show much more variation than the underlying protein backbone. The degree of sequence motif conservation varies greatly among pseudo-symmetrical protein structures, so that some of them show little or no sequence symmetry obvious to the casual observer. Different models have been put forward to explain the precise role of gene duplication and fusion on their tertiary and quaternary structures, but all of these suggest that a similar route can be exploited for artificial protein design to yield symmetrical proteins and complexes [2]. Such structures are attractive scaffolds for metal-coordinating complexes, synthesizing inorganic nanoparticles, and for therapeutic applications. Here we describe recent progress in the design of proteins following different methods such as sequence-based approach and 3D modelling, schematically represented in Fig. 1.

## 2 Computational protein design

Initial designs of repeat proteins focused on the idea that a consensus repeat, derived from a natural protein, may be able to fold into a similar but more regular structure than the template (Fig. 1). It was generally found however that the design of repeat proteins from sequence consensus alone does not always result in stably folded structures [7]. Structural features of the model must generally be taken into consideration to produce accurate designs of predefined architectures, optimizing interactions between residues and the packing of the hydrophobic core, but exploiting the information in a molecular model significantly complicates the design process [8]. Computational protein design (CPD) starts from a chosen fold with the desired properties, and decorating this backbone with side-chains. Multiple rounds of sequence selection, optimization, and scoring are performed to search for sequences that adopt the fold stably, and match any other applied constraints (Fig. 1). These constraints may be geometrical, and may involve interactions with a binding partner so that the designed protein possesses a desired binding specificity. Since the sequence space is huge, even for small proteins, one advantage of repeat protein design is the sequence constraints, which greatly reduce the search space. For example, if each of the twenty normal residues can occur at each position of a 200 residue protein, then there are $20^{200}$ possible sequences. However, if the protein is constrained to be symmetrical with five identical subdomains, then the number of possible sequences is reduced to $20^{40}$, and this reduction in complexity allows much faster optimization. Currently, a variety of software packages, including ORBIT [9], OSPREY [10], and Rosetta [11], [12] are available for the design or redesign of proteins.

Dahiyat and Mayo generated a cyclic design strategy named ORBIT (Optimization of Rotamers By Iterative Techniques). During the design step, all possible rotamers are mapped on a backbone (Fig. 1) Subsequent low scoring rotamers are eliminated via a Dead-End Elimination (DEE) algorithm [13], thereby reducing the conformational space. Following single residues, DEE is also applied on rotamer pairs until an optimal sequence is found. During the final design step rotamers of randomly selected residues are probed in a Monte Carlo based search approach. The final obtained sequence is experimentally validated and the results are linked back to the initial design to allow a further optimization of the design [9]. ORBIT was demonstrated by re-engineering a small protein domain to fold without its natural zinc ion. Zinc-finger proteins do not fold without zinc, but in its presence they adopt a ββα fold. Dahiyat and colleagues successfully redesigned a zinc-finger using ORBIT to give it a small hydrophobic core in place of the natural zinc-binding residues, allowing it to fold in a metal-free state [14].

In contrast to ORBIT, OSPREY (Open Source Protein Redesign for You) focuses on the redesign of existing proteins. After defining a region of interest, such as a protein core or interface, mutations are applied to improve the packing or to add extra functionality. A continuous rotamer library is then used to find lower energy states by applying multiple algorithms including DEE [10]. OSPREY has been used successfully for the design of inhibitors, to alter specificity, and to predict drug resistance [15], [16], [17].

Both ORBIT and OSPREY focus on the redesign and optimization of proteins. Interactions within the model are optimized in multiple cycles, using DEE to prune unfavorable residue types from each position, while keeping the backbone fixed. Rosetta, originally developed as software to fold proteins ab initio, offers a wider range of tools for the modeller. To predict the fold of a given amino acid sequence, models are obtained by comparing short regions with a structural database, and then scored against an energy function. Rosetta has expanded to allow not only the redesign of proteins, but also de novo design, docking, and protein-DNA interactions. This versatility, together with its large user base and continuous improvements, makes the Rosetta suite an extremely useful design tool [18], [19].

## 3 Solenoids

**Fig. 2:**
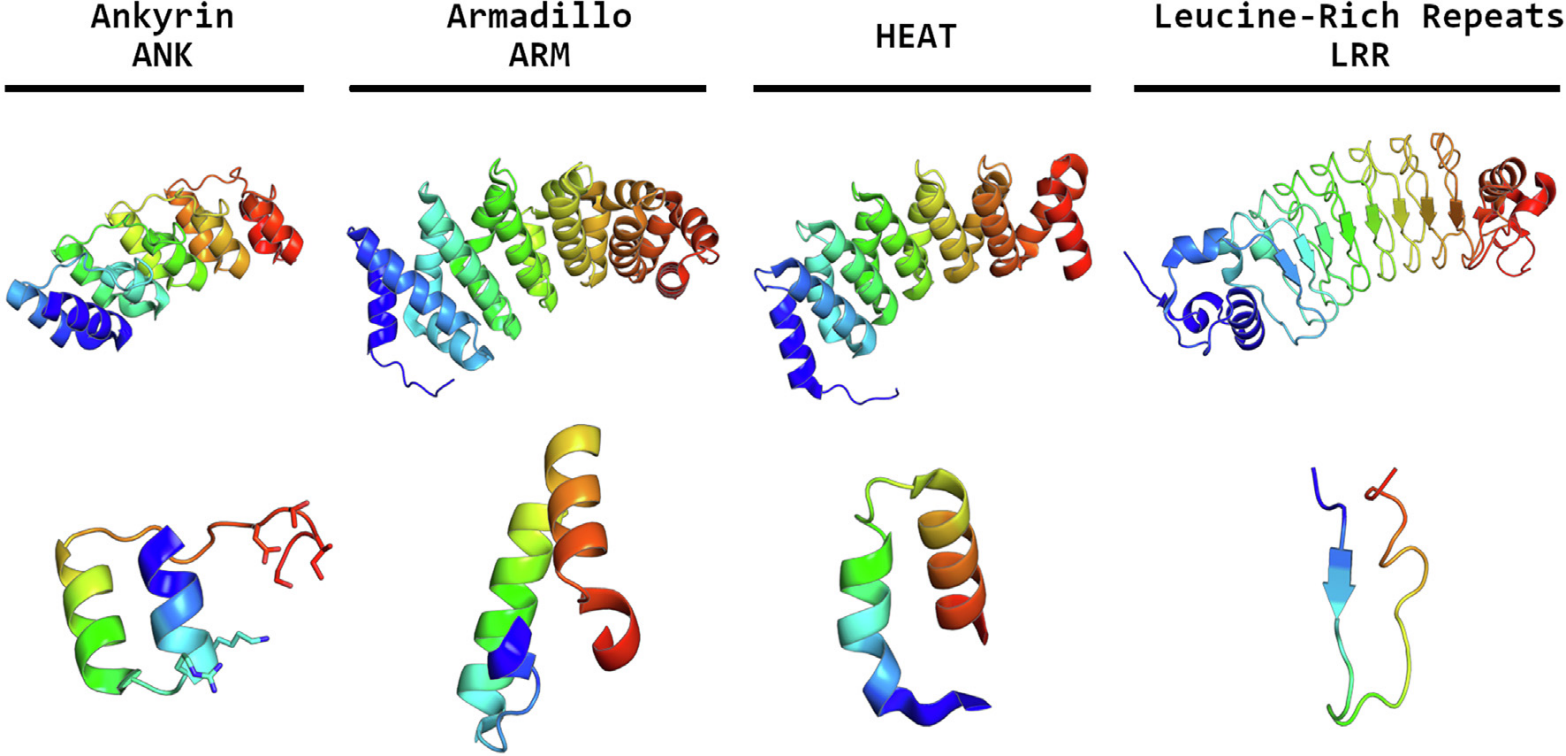
Designer repeat proteins with a solenoid symmetry. Each protein consists of multiple identical repeats, originating from consensus design, which are shown underneath. Structures depicted are the ANK repeat (PDB code: 2BKG), ARM repeat (PDB code: 4V3Q), HEAT repeat (PDB code: 3LTJ), and the LRR protein (PDB code: 3RFJ). In addition, the six variable residues responsible for target binding in ANK repeats are shown in sticks.

Symmetric proteins can be divided into two main categories, with open or closed symmetry. Closed symmetry occurs when structural motifs are repeated in such a fashion that the polypeptide chain termini are located close in space. On the other hand, if neighboring repeats of the structural motif are related by a translation in space, with or without a rotation, then an open-ended structure will result. Solenoidal proteins are built from repeated structural motifs that assemble as a continuous superhelix. They are very common, and include ankyrin (ANK), armadillo (ARM), and HEAT repeats (Fig. 2). Each motif of about forty amino acid residues is relatively well conserved within each protein, and repeated up to thirty times. Usually these motifs form two or three α-helices connected by a small loop, but in the leucine-rich repeat (LRR) proteins each repeat also forms a β-strand. LRR proteins produce horseshoe-shaped loops with different curvature, the β-strand forming an incomplete barrel-like sheet on the inside of the loop. Alignment of the motifs within these structures suggests that conserved residues stabilize the overall fold, and other residues are responsible for ligand interactions. Similar to antibodies, it should be possible to build almost identical proteins with different binding properties. Artificial DARPins (Designed Ankyrin Repeat Proteins) were built from the consensus ANK repeat sequence [20] and the same method was used to create αRep proteins from HEAT repeats [21] (Fig. 1).

Of the 33 amino acid residues in the consensus ANK repeat, six are not conserved. Four of these residues form a protruding loop between adjacent repeats, while the other two are located at the beginning of the second α-helix (Fig. 2). As these variable residues are responsible for recognition of specific targets, a combinatorial DARPin library can be created to facilitate screening for desired binding properties by selection methods such as phage display. This technology shows enormous promise in the creation of antibody-mimetic protein-based drugs. For example, wet age-related macular degeneration (wAMD) can be treated by anti-angiogenic agents, and a DARPin (called abicipar) successfully completed phase III clinical trials in 2018 [22]. Unfortunately it was not finally approved by the FDA due to its side-effect profile [23], but other DARPin-based drug are still in different phases of clinical trial studies [24]. Non-medical applications of DARPins include co-crystallization of proteins by stabilizing flexible protein loops and the study of protein regulation. DARPins are also of interest as biosensors or diagnostic detection systems [20].

Rämisch et al. adopted a computational approach to optimize a consensus-based design of LRR domains with a predefined, desired curvature. After selecting appropriate tandem repeats, multiple cycles of mutation and interface optimization were then performed without including any linker region between the repeats. Subsequently, a gene was created to express multiple copies of the optimized motif, linked by a short peptide, resulting in a repeat protein with the required shape [25]. A further step in the design of LRR proteins was to make a number of self-associating motifs with different curvatures, and also junction sequences that could sit between motifs of different types. Park et al. created a small library of self-compatible LRR repeats and showed these could be readily combined using the junction repeats to build very easily custom-designed LRR proteins with varying curvature [26].

**Fig. 3:**
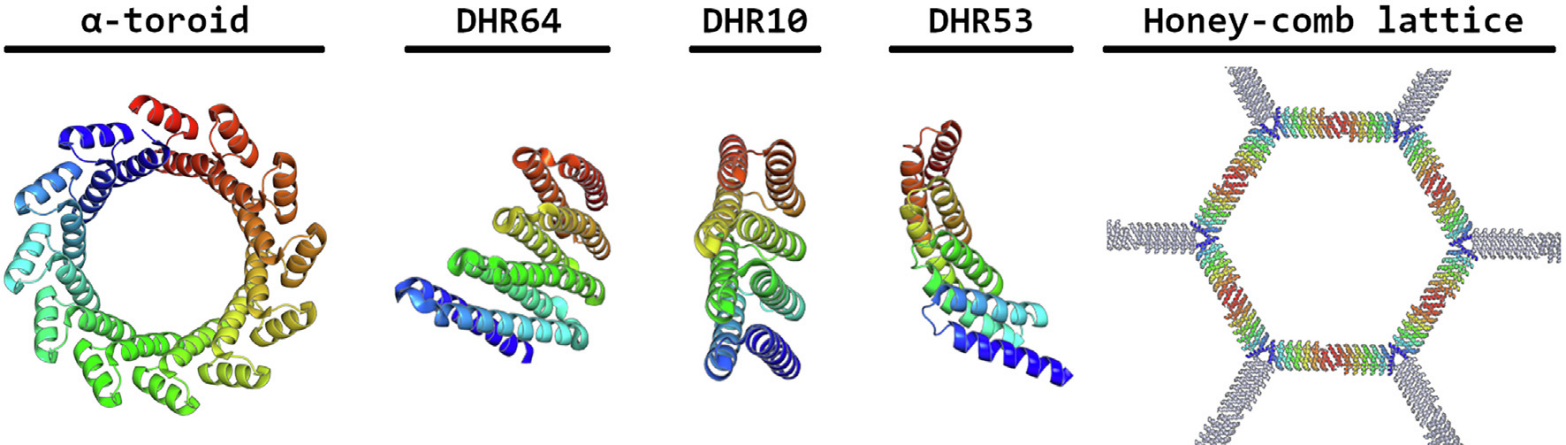
Computationally aided structures based on solenoid repeats. Doyle et al. designed the first closed left-handed α-toroid structure (PDB code: 5BYO). Around the same time Brunette et al. sampled a fold not explored before by nature, ranging from left and right-turning solenoids to untwisted solenoids (PDB code: 5CWM, 5CWG, and 5CWK). Furthermore, DHR10 was demonstrated to interact with inorganic lattices due to incorporation of carboxyl residues. Next, two-fold and three-fold symmetric interfaces were integrated at the DHR10 termini, resulting in honeycomb assemblies on inorganic lattices (Model: DHR10mica7H_honeycomb from Pyles et al.).

In contrast to Park et al., the group of Bradley used only geometric constraints to design α-helical tandem repeats with the Rosetta software package, and avoided the use of natural protein structures and sequences [27]. Left-handed α-helical repeats were aligned in a plane to form closed toroids, an architecture explicitly chosen because it had not been reported before. Successful designs were achieved with proteins having 3, 6, 9 or 12 repeats, and five crystal structures were determined, showing close agreement with the computer models, although closer for the smaller proteins [27]. Later the same group produced a structure with similar architecture, built from 24 repeats of a 33 residue motif, each repeat forming two amphipathic left-handed α-helices [28]. The protein forms a ring with internal and external diameters of 6 nm and 10 nm respectively (Fig. 3). Notably polypeptides carrying 6, 8 or 12 repeats of the same motif could also assemble non-covalently into a 24-repeat structure. Since these truncated proteins form regular oligomers they were subsequently used to display other proteins such as scFv domains or SpyCatcher in order to create higher affinity complexes with chosen targets [28].

Similar to the design strategy of Doyle et al., Brunette and colleagues created DHRs (designed helical repeat proteins) based on a repeated helix-loop-helix-loop motif using an automatic Rosetta-based strategy, without limiting themselves to naturally occurring folds or sequences (Fig. 1) [29]. Nearly 6,000 non-natural sequences were generated containing varying helix and loop lengths, and 83 of these were experimentally characterized. Crystal structures were solved for some of the designs, confirming that they spanned a wide architectural range, from left- and right-twisted solenoids to flat linear repeats and toroids, including folds not seen in nature (Fig. 3) [29]. The authors noted that their results indicate a very large number of repeat protein sequences may be able to form stable structures.

Many natural proteins function through interactions with inorganic surfaces. Ice-binding proteins for example prevent water from freezing by coating ice crystals to prevent them from growing. Inspired by these proteins, and the ability of repeat proteins to match the lattice structure of minerals, Pyle and colleagues attempted to design proteins that complemented a surface of mica crystals, both electronically and structurally [30]. Potassium ions form an ordered sub-lattice on the 001 face of muscovite mica, with the shortest distance between them being 5.2Å. The group reasoned that a flat, repeat protein structure with the same spacing could interact with the regularly positioned metal ions through surface carboxyl residues. The DHR10 motif, described earlier by Brunette et al., proved highly suitable as a framework. DHR10-mica was created from DHR10 by placing glutamate residues at suitable positions, and DHR10-mica18 was made by joining 18 copies in a single polypeptide. AFM showed this protein could bind tightly to mica as expected, and a further elaboration of the design by adding a dimerization and trimerization domain allowed the group to build honeycomb structures on the mica surface as well (Fig. 3). This ability to build different complexes on inorganic lattices very simply from a basic motif shows there are many possibilities for applications at the boundary between biological and inorganic systems [30].

## 4 Globular symmetrical proteins

Tandem repeat proteins derived from gene duplication and fusion events are not limited to solenoidal folds, and in fact tandem repeats are often arranged around a central axis of rotational pseudo-symmetry, resulting in a toroid architecture. Work with three toroidal folds is discussed in this section.

### 4.1 TIM-barrel protein

Triose phosphate isomerase (TIM) is a much studied protein, used as a model system to understand the fundamental principles of enzymes, and memorably described as “catalytically perfect” [31]. There was therefore considerable interest in determining this protein structure even in the very early days of protein crystallography, and it was the first example of a protein model with an α/β-barrel made from eight consecutive repeats, each consisting of a β-strand followed by an α-helix [32]. The strands form a central β-barrel, with the helices on the outside, so the fold was named the TIM barrel.

**Fig. 4:**
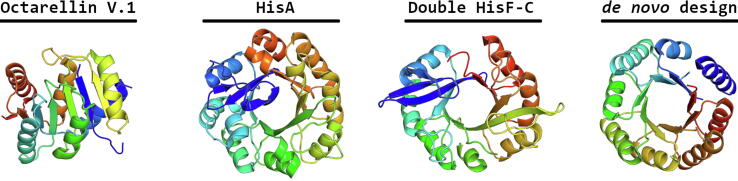
Evolution of the design of TIM-barrels. Starting by the first design attempts, the final Octarellin model (PDB code: 5BOP) is shown. Next is HisA (PDB code: 1QO2), which aided in the discovery that TIM-barrels originate from half-barrels and the design of a half-barrel from HisF (PDB code: 2W6R). Finally the first de novo design by Huang et al. (PDB code: 5BVL).

The first design of an artificial, perfectly symmetrical α/β-barrel, by Martial and co-workers, was named Octarellin [33]. They assumed that a sequence with the correct α/β-packing that satisfies the predicted length and matching interactions of secondary structure elements would result in a well folded TIM-barrel. Characterization of Octarellin by circular dichroism (CD) spectroscopy showed the expected level of helical structure, but the protein did not achieve a stable fold. The group tried to improve the design by switching to a four-fold symmetry, and optimizing the β-sheets using Rosetta-based design strategies. One of the final models, Octarellin VI, had improved stability but solubility remained low. Further optimization yielded Octarellin V.1, whose tertiary structure resembles a Rossmann-like fold more than the intended TIM-barrel (Fig. 4) [33], [34], [35], [36]. In 2003 the Martial group succeeded in designing a de novo TIM-barrel, but low solubility prevented the structure from being determined [37]. This work was followed in 2015 by the Rosetta-based design of Symmetrin, another de novo model with four-fold rather than eight-fold symmetry. Nagarajan and colleagues selected four variants of this sequence for experimental validation, one of which proved insoluble. The remainder showed cooperative folding transitions and secondary structure by CD spectroscopy, but NMR analysis showed none of them possessed a well-defined defined tertiary structure [38].

Some years before Octarellin VI and Symmetrin it had been found that two similar TIM-barrel enzymes have apparently both evolved from a common half-barrel [39]. The enzymes, phosphoribosylformimino-5-aminoimidazol carboxamide ribotide isomerase (HisA) and cyclase-producing d-*erythro*-imidazole-glycerolphosphate (HisF), can be structurally aligned with an RMSD of less than 2Ångström, overlaying conserved residues in the two sequences. The hypothesis of a common ancestor was tested by screening multiple mutants of both enzymes, as well as expressing stable half-barrels and chimeric barrels (Fig. 4). The ancestral half-barrel was probably able to form either dimers or tetramers in order to form a complete hydrophobic core, leading to the modern monomeric TIM-barrels by gene duplication and fusion [40], [41], [42], [43].

The first properly folded, computationally designed TIM-barrel was produced by the groups of Baker, Höcker, and Velasco in 2016 [44]. Like Nagarajan and colleagues, they noted that the simplest topology for a TIM barrel involves four identical repeats, not eight, because of the alternating pleat of paired beta-strands. In contrast to earlier design protocols, Huang and co-workers also applied strict criteria regarding the backbone hydrogen bonding of each model in order to ensure the register of the β-strands formed was correctly and stably established. Additional constraints were added to ensure optimal connection between the β-strands and a stable hydrogen-bond network within the loops. Subsequently, de novo fragment assembly calculations were performed with Rosetta to obtain backbone models with varying loop and helix lengths [45], [46]. From these models, one was chosen that was low in energy and yielded a closed barrel structure with an extensive hydrogen-bond network. Ensembles of sequences were mapped on the chosen backbone via iterative cycles, probing all possible side chain orientations using all-atom energy minimization. Finally, the 22 lowest energy designs were experimentally tested, and five showed cooperative folding. To promote crystallization, the protein was circularly permuted and cysteine residues were incorporated. The resulting crystal structure was solved, demonstrating that this de novo four-fold symmetric TIM barrel was stably and correctly folded as well as soluble (Fig. 4) [44].

### 4.2 β-Trefoil protein

**Fig. 5:**
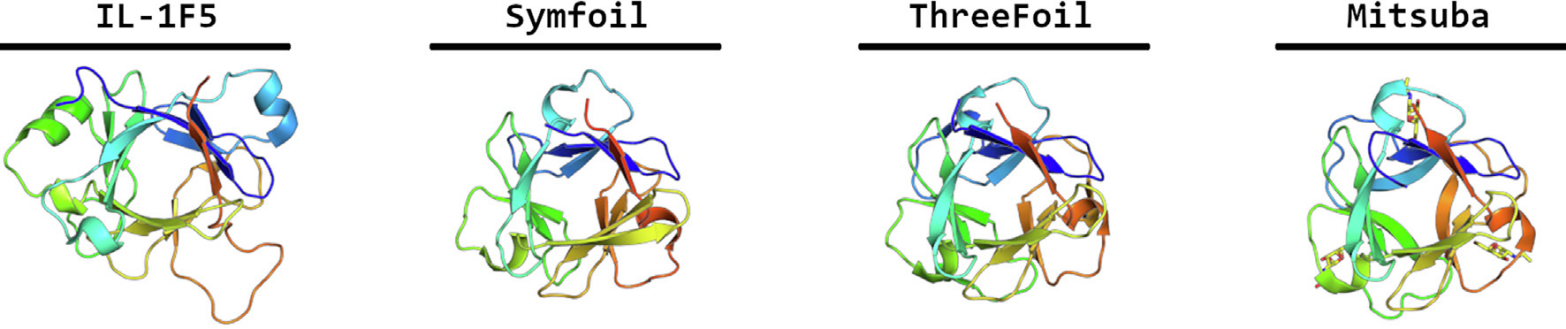
Evolution of designed β-trefoils. First protein shown is a natural motif from interleuking family (PDB code: 1MD6), followed by the Symfoil and ThreeFoil designs (PDB code: 4F34 and 3PG0). Last shown is Mitsuba, the first designed protein with trefoil motif that can recognize certain cancer cells (PDB code: 5XG5).

β-trefoil proteins are very widely distributed across all three Kingdoms. Their fold is characterized by six β-hairpins, three of which form a small β-barrel, while the remainder form a triangular cap (Fig. 5) [47]. Although the hydrophobic regions are conserved to some degree among different families with this fold, the general level of conservation is low. The extensive loops vary greatly in length and sequence, giving large unique protein surfaces suitable for recognition of specific partners. β-trefoils are therefore suited to a variety of functions, and play roles in protease inhibition, initiation of immune responses, and control of mitogenic and angiogenic activity. Lee and Blaber sought to understand the evolution and folding of trefoils by deconstruction of human fibroblast growth factor-1 [48]. Multiple rounds of rational design of secondary structure elements were performed while conserving the hydrophobic core, which gradually improved the symmetry and stability, resulting in an artificial β-trefoil protein named Symfoil (Fig. 5) [48]. Subsequently truncated versions were also expressed and shown to fold. The single bladed Symfoil adopted a trimeric form in solution without any trace of the monomer. The two-bladed Symfoil also trimerized, folding into two domain-swapped β-trefoil motifs, with one polypeptide chain linking the trefoils. This conservation of architecture as the protein is subjected to extensive mutation supports the notion of a common ancestor of all β-trefoil proteins, as suggested earlier by Ponting and Russell [49].

Contemporaneously with Lee and Blaber, Broom and colleagues in the group of Meiering attempted the rational design of a three-fold symmetric β-trefoil, starting with a search for natural trefoils with the highest levels of internal symmetry [50]. Using a carbohydrate binding module from the ricin family, they created a perfectly three-fold symmetric β-trefoil named ThreeFoil through a combination of rational and consensus design (Fig. 5). Since the ligand binding sites within each motif were conserved during the design, Threefoil was able to bind three suitable sugar molecules at equivalent positions on the fold [50]. Although Threefoil proved to be stable, truncated versions with one or two repeated motifs instead of three were unable to fold.

Trefoil motifs make interesting templates for artificial proteins partly because they are relatively small, but also because they bind a variety of ligands with potential clinical or technological uses. Recently, a novel trefoil lectin called MytiLec was discovered that not only recognizes α-D-galactose, but also shows cytotoxic activity to certain cancer cell types [51]. Terada and colleagues sought to combine Ancestral Sequence Reconstruction (ASR) with CPD to obtain a three-fold symmetric variant of the natural protein [52]. They followed the reverse engineering evolution (RE_3_volutionary) protein design strategy, originally developed to create a propeller protein described in detail below (Fig. 1). MytiLec forms part of a small and unusual subfamily of galactose binding trefoils, without the classical QxW motif of the ricin-like proteins. The three subdomains of the protein are also poorly conserved in places due to the dimerization interface, which weakens the three-fold pseudo-symmetry. As a result, evolutionary reconstruction created a model with a large central cavity that appeared unlikely to fold stably. Comparison with the earlier Threefoil structure suggested improvements in the linker region between motifs, and as a result a stable protein named Mitsuba (Japanese for trefoil) was successfully expressed. Like Threefoil, Mitsuba is able to bind three ligands at equivalent sites. The crystal structure of MytiLec closely matches the natural template, but is monomeric, so that it is unable to haemagglutinate red cells unlike the parental protein (Fig. 5). Cytotoxicity is also lost towards all cell types [52].

### 4.3 β-Propeller protein

The β-propeller fold consists of a circularly arranged set of β-sheets around a central pore. Each β-sheet, commonly referred to as a ‘blade’, is comprised of four anti-parallel β-strands [53]. Natural propeller proteins have between four to ten blades, usually with the N- and C-termini close within one blade. It is often observed that the C-terminal β-strand completes the last β-sheet, an arrangement referred to as a Velcro strap or closure [54]. Another characteristic of β-propellers is the pronounced sequence variation within and between families, suggesting their innate stability allows them to support enormous diversification. Propellers often act as interaction hubs, as regulators for cellular functions, and therefore related to some clinical conditions [55], [56]. Various sub-families have arisen, including the widespread WD40 proteins, whose name stems from a characteristic repeated Trp-Asp motif at the end of the third β-strand.

The first attempts to create symmetrical β-propeller proteins used members of the WD40 family as templates. Inspired by the earlier consensus design of α-helical repeat proteins, Nikkhah et al. attempted to design the first artificial β-propeller. An idealized WD repeat was obtained by manual editing of a consensus sequence obtained from seven-bladed templates, and constructs consisting of four to ten tandem copies of this sequence were tested experimentally. Proteins with between four and eight repeats could be expressed and purified, but characterization showed the proteins formed molten globules and were prone to aggregation [7].

**Fig. 6:**
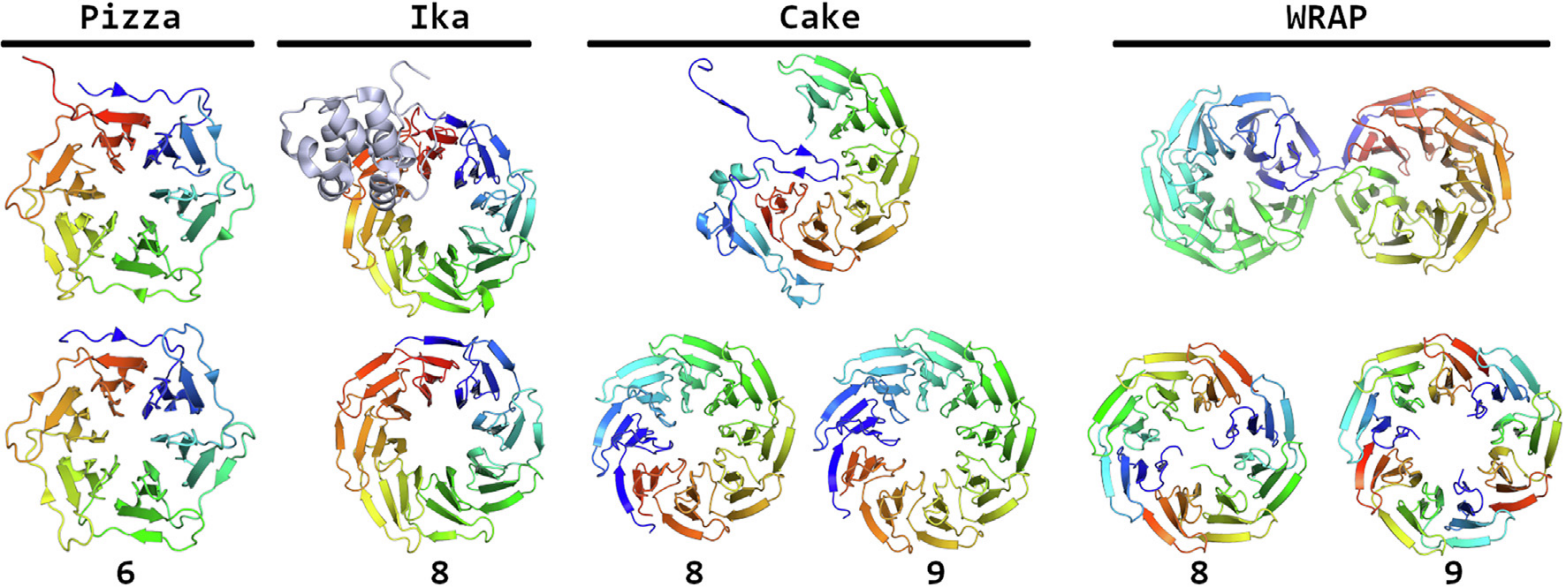
Comparison of designed β-propellers with their original template. The number of blades present in the designed propellers is shown underneath. First shown is the β-propeller from a kinase domain, which was used as a template for the design of Pizza (PDB code: 1RWL and 3WW9, respectively). Next is the eight-bladed template used for the design of Tako, followed by Ika (2OVP and 6G6P). More recently, a partially disordered quinoprotein was used to design a fully symmetric propeller, however, it led to two propellers with a structural plasticity, namely Cake8 and Cake9 (PDB code: 3HXJ, 6TJG, and 6TJH). Finally, the recent evolved fourteen-bladed WD40 propeller that via truncation led to an eight-bladed and nine-bladed WRAP propellers (PDB code: 2YMU, 6R5X and 6R5Z).

Eight years later, Voet et al. succeeded in designing the first artificial β-propeller [57]. From a set of 174 β-propellers, one protein was chosen as a template due to its notably pseudo-symmetrical backbone, and relatively high sequence conservation over all the blades. All six blades from this model were structurally and sequentially aligned and used as input for ASR via the FastML web server [58]. At the same time a complete six-fold symmetric backbone was generated via RosettaDock [59], and a Rosetta-based algorithm was used to map all putative sequences on the best scoring backbone model. The design strategy was named RE_3_volutionary (REverse Engineering Evolution) protein design, as both ASR and CPD are utilized. The top ranking results from the Rosetta scoring function were carefully inspected, and the most promising sequence was experimentally characterized (Fig. 1). This protein, called Pizza6, expressed extremely well in *E. coli* as a monomeric β-propeller (Fig. 6) [57]. Two-bladed and three-bladed variants expressed equally well, oligomerizing into a trimer and dimer, respectively, to recreate the six-bladed β-propeller. This self-assembling property was used to make fusion constructs which fold into symmetric cages that can be further functionalized [60] and used for example for vaccine design [61]. The same design strategy was also applied to an eight-bladed WD40 β-propeller, to create Tako8. Although Tako8 expressed at high levels, the protein became less stable in the absence of salt or at low pH. Truncated versions of Tako8 did not express or assemble, unlike Pizza6 [62]. The design was further optimized by combining an exact deterministic CPD tool with ToulBar2 [63], [64], [65]. The backbone of two adjacent Tako8 blades was relaxed while the CPD tool was utilized to find the sequence adopting this fold with global minimum energy. This was done by checking each rotamer of each amino acid at every position throughout the double blade. Four residues were conserved as these are crucial for the hydrogen-bond network stabilizing the packing of each blade. Next, four-fold symmetry constraints were applied to the two blades to recreate a full eight-bladed β-propeller, called Ika8 (Fig. 6). In contrast to Tako8, Ika8 was able to express and assemble even when truncated to two or four blades [62].

Mylemans et al. attempted to recreate a symmetric β-propeller based on a naturally occurring incomplete propeller domain. By following the RE_3_ volutionary protein design approach with a 9-fold symmetric backbone, an idealized sequence was obtained, named Cake9 [66]. Surprisingly, truncated variants were found to fold as β-propellers with either eight or nine blades (Fig. 6). This structural plasticity was also observed by Afanasieva et al., who focused on a relatively recently evolved seven-bladed β-propeller with a high level of internal symmetry. Following truncation into fragments consisting of three or four repeats, the constructs gave rise to eight-bladed and nine-bladed propellers, but the internal symmetry was not completely maintained (Fig. 6) [67]. The observed plasticity found for some β-propellers may hint at the evolutionary origin of these proteins, and how nature may re-use flexible protein parts for different functions [68].

While previously described studies focused on the design of fully symmetric propellers, the group of Tawfik sought to determine the evolutionary pathway of the five-bladed β-propeller tachylectin. They started by expressing a variety of two-bladed circular permutants derived from the natural β-propeller, revealing homo-pentamers with twice the size of the original protein [69]. Although at first these proteins were poorly soluble and prone to aggregation, directed evolution yielded improved proteins, allowing the crystal structure to be determined, confirming a double five-bladed propeller structure with domain-swapped strands [70]. These experiments confirmed that simple segments, possibly even smaller than one blade, may eventually result in different blades that fold with a propeller architecture, if subject to multiple consecutive cycles of duplication, fusion, and diversification events.

To demonstrate this hypothesis, ASR was applied to tachylectin-2, generating a library of single blades that was subsequently subjected to directed evolution and screened for glycoprotein binding. This led to the identification of a single blade able to assemble into a 5-fold symmetrical propeller architecture. One conclusion from the work is that circular permutation from non-Velcro to Velcro forms of a tandemly repeated protein may occur at a late stage in the evolutionary process, and that folding and stability drive the selection following duplication and fusion events [71].

### 4.4 Ferredoxin fold

Ferredoxins (FDs) are pseudo-two-fold symmetric, and thought to be among the earliest proteins to evolve as they are found in all three domains of life [72]. Bacterial FD proteins generally coordinate two iron-sulfur (Fe-S) clusters, and often bind an additional zinc ion at a separate site in the N-terminal domain. Research by the group of Gomes demonstrated that loss of the zinc binding site or N-terminal domain reduces thermal stability, although FDs naturally devoid of zinc appear to be more stable than than the zinc-containing variants. This stability arises from hydrophobic residues which hold the N-terminal β-strands to the core more effectively than a solvent-inaccessible zinc binding site [73], in a manner reminiscent of the redesigned zinc-finger of Dahiyat and colleagues [14].

Similar to the studies by the Tawfik group, Falkowski and colleagues sought to redesign FD proteins to help understand their evolutionary origin. Phylogenetic analysis suggested that ancient FDs may have shown up to 60% sequence identity between their two halves. This similarity was mostly found for the inner part of the protein, while the outer layers were more distinct, allowing the protein optimal performance as an electron shuttle. Based on their consensus sequence, ASR was performed to obtain two-fold symmetric proteins based on the C-terminus, N-terminus, and both termini. All three proteins expressed well and demonstrated successful coordination of two Fe-S clusters. Furthermore, the proteins were able to undergo reversible oxidation and reduction and demonstrated electron transfer properties [74].

## 5 Summary and outlook

In the last decade, many symmetrical proteins have been designed and developed based on naturally occurring pseudo-symmetrical templates. These designer proteins have helped us understand possible pathways of protein evolution, as well as develop novel CPD approaches to obtain improved proteins. Although protein design has made tremendous progress in the last few decades, its commercial applications are still limited. ANK repeats built using consensus sequences (DARPins) have a variety of clinical applications, and several have entered advanced trials, one even passing phase III recently. DARPins can do much that antibodies can do, but are simpler and cheaper to produce. The redesign of existing proteins can lead to improved functions, such as the increased thermostability of a phytase to serve as a feed additive [75]. In addition, redesign can allow us to incorporate new functions, or remove unwanted ones. The redesigned β-trefoil, Mitsuba, for example retains recognition of specific cancer cells but does not haemagglutinate red blood cells [52]. β-propellers based on Pizza protein were developed as enzymes that can carry out either a stereo-specific Diels-Alder reaction or hydrolysis [76], [77]. Although rational design is not limited to the reworking of natural proteins, and produces stable designs much more reliably than consensus alone, repeat protein design is not yet trivial. Although the design of a mica-binding protein was a great success [30], incorporating specific metal-coordinating interactions into proteins has also proved difficult, so that variants of ferritin have proved much more popular for biomineralization than de novo templates [78]. The Pizza protein has also been shown to epitaxially grow a mineral crystal on a protein surface, leading to a nanocrystal of cadmium chloride [79]. In contrast, CPD allows the design of artificial proteins that grow large complexes in the presence of metal ions, which gives a simple means of control over the assembly. In addition, symmetric inorganic complexes such as polyoxometalates can be coordinated by designer proteins as well, potentially leading to hybrid biomaterials combining the advances of biocompatbilility with the catalytic and electronic properties of the inorganic molecules [80]. CPD continues to improve, allowing new and larger structures to be created for applications ranging from bio-catalysis and bio-templating to clinical testing and drug use. As stable structures that can be designed relatively easily, repeat proteins are expected to play a large role in these fields in the future.

## Declaration of Competing Interest

The authors declare that they have no known competing financial interests or personal relationships that could have appeared to influence the work reported in this paper.
